# Standardized reporting and quantification of whole-body MRI findings in children with chronic non-bacterial osteomyelitis treated with pamidronate

**DOI:** 10.1186/s12969-022-00746-y

**Published:** 2022-10-01

**Authors:** C. M. Andreasen, R. F. Klicman, T. Herlin, E. M. Hauge, A. G. Jurik

**Affiliations:** 1 Department of Rheumatology, Aarhus University Hospital, Aarhus N, Denmark; 2 Department of Clinical Medicine, Aarhus University, Incuba Skejby, Aarhus N, Denmark; 3 Department of Medicine, Rheumatology, Gødstrup Hospital, Herning, Denmark; 4 Department of Pediatrics and Adolescent Medicine, Aarhus University Hospital, Aarhus N, Denmark; 5 Department of Radiology, Aarhus University Hospital, Aarhus N, Denmark

**Keywords:** Whole body magnetic resonance imaging, Standardized reporting, Radiological outcome measure, Chronic non-bacterial osteomyelitis, Chronic recurrent multifocal osteomyelitis, Case-study, Diphosphonates, Pamidronate

## Abstract

**Objectives:**

The objectives were to assess changes in radiological disease activity in children with chronic non-bacterial osteomyelitis (CNO) receiving pamidronate therapy and to test a modified radiological index for non-bacterial osteitis (mRINBO) in CNO. mRINBO was used for standardized reporting and quantification of whole-body MRI (WBMRI) findings resulting in an individual summary patient score.

**Methods:**

WBMRI was retrospectively assessed in 18 children with CNO at baseline and after receiving pamidronate therapy for one year. Parameters of interest were: number and anatomic site of radiologically active bone lesions (RAL), size of RAL, extramedullary affection, spinal involvement and changes in mRINBO, which includes both the number and maximal size of RAL (RALmax) in addition to extramedullary and chronic changes.

**Results:**

At the time of diagnosis, the mean age of the children was 9.8 (sd, 8.7–10.9) years and 11/18 were females. The number of RALs per patient decreased from median [interquartile range] 4.5 [3–8] to 3 [2–5] RALs per patient (*p* = 0.02) and extramedullary inflammatory changes regressed. Sixty-one percent of all RALs occurring at baseline resolved and three children became without active inflammatory lesions by WBMRI. The median size of RALs did not change when taking new lesions occurring in 7/18 children into account, but RALmax decreased significantly from 39 [29–45] mm at baseline to 28 [20–40] mm (*p* < 0.01) at year-one with a concomitant decrease of mRINBO from a median of 5 [4–7] to 4 [3–5] (*p* = 0.05).

**Conclusions:**

Pamidronate therapy resulted in a decrease of mRINBO from baseline to year one. mRINBO may be a potential scoring method to quantify changes in radiological disease activity in children with CNO. However, further studies are needed to test feasibility and validity of mRINBO.

**Supplementary Information:**

The online version contains supplementary material available at 10.1186/s12969-022-00746-y.

## Background

Chronic non-bacterial osteomyelitis (CNO) is a skeletal disorder in children and adolescents. CNO is characterized by sterile and often multifocal inflammatory bone lesions [1] with a predilection for the metaphyses of the long tubular bones in the lower extremities, the axial skeleton and the clavicles. During the disease course bone lesions can vanish or persist and bone lesions may occur at new sites [2–6]. Children with CNO present clinically with musculoskeletal complaints such as pain and swelling at the skeletal sites involved and spinal lesions can lead to vertebral fractures and risk of kyphoscoliosis [7, 8]. However, radiologically active lesions may be asymptomatic [2, 3, 9].

In CNO, MRI is useful to assess active and chronic bone inflammation, synovitis and adjacent soft-tissue edema. Whole-body MRI (WBMRI) is increasingly being used initially at time of diagnosis to identify clinical silent lesions and to detect multifocal bone lesions at typical locations. Follow-up scans are used to detect complications, new lesions and clinically silent lesions when monitoring the disease during treatment [2, 5, 10–14]. Due to rather variable CNO disease courses with vanishing active lesions and potential occurrence of new active or chronic lesions a global assessment and reporting of disease activity is important. The purpose of developing a standardize radiological outcome measure in CNO is to develop a tool that can be used to describe changes in radiological disease activity both in clinical studies and to monitor disease activity in a daily clinical setting.

A radiological index for non-bacterial osteitis (RINBO) has been suggested for standardized reporting of WBMRI in children with CNO [2]. RINBO includes four parameters of interest: number and maximal size of radiological active lesions, extramedullary changes and spine involvement encompassing both active and chronic lesions in the form of osseous hyperostosis and vertebral deformity (Table 1). A RINBO scoring is easy to perform and results in an individual summary patient score describing radiological disease activity. However, it has not been evaluated in longitudinal studies.

**Table 1:** The original and the modified radiologic index for standardized reporting of whole-body MRI in patients with chronic non-bacterial osteomyelitis

Parameter of interest	Criterion RINBO	Criterion mRINBO	Value
No of patient’s radiological active lesions			
Unifocal	1	1	1
Paucifocal	2–4	2–4	2
Multifocal	≥ 5	≥ 5	3
Maximum size of patients’ radiological active lesion			
Minor	< 10 mm	< 20 mm	1
Average	10–100 mm	20–50 mm	2
Major	> 100 mm	> 50 mm	3
Extramedullary affection			
Acute	Periostal reaction and/or soft tissue edema	Periostal reaction and/or soft tissue edema	1
Chronic	Hyperostosis	Hyperostosis	1
Spinal involvement			
Acute	Radiologically active vertebral lesion	Radiologically active vertebral lesion	1
Chronic	CNO related vertebral body deformation	CNO related vertebral body deformation	1
**Maximum index point**			**10**

*RINBO* Radiologic index for chronic non-bacterial osteomyelitis (21), *mRINBO* Modified RINBO score with new definitions of the maximum size of patients’ radiological active lesion

based on the current sizes and exclusion of radiological active lesions in the metatarsal bones

Treatment of CNO aims to reduce inflammation, osteolytic progression, disability and pain. The therapeutic approach to CNO is based on low level of evidence. Several treatment algorithms have been suggested [6, 15]. Non-steroidal anti-inflammatory drugs (NSAIDs) are recommended as first-line therapy [3, 16]. Children with multiple lesions or spinal involvement are considered to have more challenging disease courses [6, 17], necessitating treatment with second-line pharmaceuticals [6, 18]. In cases-series, the amino bisphosphonate pamidronate has been reported to be effective in improving clinical and radiological disease activity [6, 8, 19–29] while other studies have suggested treatment with biological disease modifying anti-rheumatic drugs (bDMARDS). In our institution children with symmetric multifocal or spinal bone inflammation and a poor clinical response to NSAID´s were offered second-line treatment with pamidronate [6].

The objective of this retrospective study was to test a modified radiological index for non-bacterial osteitis (mRINBO) for detecting changes in radiological disease activity by WBMRI and to assess the therapeutic effect of pamidronate in children with symmetrical multifocal or spinal bone inflammation treated with pamidronate.

## Methods

### Patient selection

**Fig. 1:**
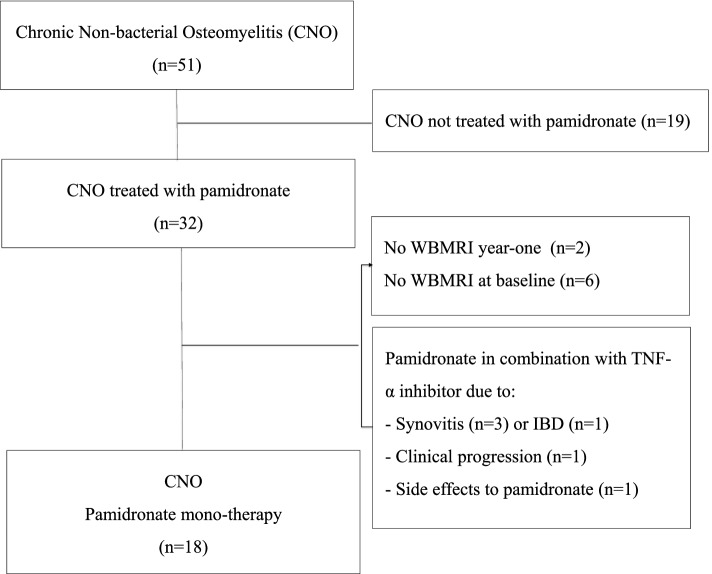
Flowchart. Fifty-one children were diagnosed with chronic non-bacterial osteomyelitis (CNO) according to the Bristol Criteria. Children with symmetrical multifocal or spinal inflammatory bone lesions and a poor clinical response to non-steroidal anti-inflammatory drugs were treated with pamidronate (*n* = 32). Reasons for study exclusion were insufficient imaging or concomitant treatment with TNF-α inhibitors. Eighteen children were included in the study. WBMRI: whole-body magnetic resonance imaging

This retrospectively selected case-series consist of 18 children (Fig. 1), representing a subgroup of 51 children previously published [6]. At time of diagnosis, the mean age of the children (*n* = 18) was 9.8 (sd, 8.7–10.9) years and 11/18 children were females. Children were all seen at the Department of Pediatrics and Adolescent Medicine, Aarhus University Hospital, Denmark, between 2007 and 2015. They were diagnosed with CNO according to the Bristol Criteria [30] and had CNO with multifocal or spinal bone inflammation and were not responding adequately to naproxen 10–20 mg/kg/day indicating initiation of treatment with pamidronate. Children were only included if a WBMRI was performed before initiation of pamidronate and following 9–18 months treatment. For one year, children were treated according to a local treatment algorithm for CNO [6] with pamidronate 1 mg/kg/day (maximum 60 mg/day) for 3 consecutive days every 3 months, first dose in the first series 0.5 mg/kg/day in addition to naproxen used pro necessitate. Children that were concomitantly treated with bDMARD, e.g. due inflammatory comorbidities, were not included (Fig. 1).

Clinical data were collected from the electronic medical records: Age, sex, time from first symptom to diagnosis, time from diagnosis to initiation of pamidronate treatment, comorbidities, concomitant medication, laboratory screening (erythrocyte sedimentation rate (ESR), HLA-B27 status) and performance of bone biopsy.

### Clinical assessment

Information on clinical disease activity was assessed from the medical records at baseline and at the visit closest to WBMRI year-one. Symptoms were defined as patient- and/or parent-reported musculoskeletal pain and/or observed swelling at the skeletal sites involved. Signs of inflammation were defined as physician-reported swelling or warmth at affected skeletal site, and elevated ESR (> 20 mm/h) attributable to CNO was used as a biochemical marker of inflammation. Clinical inactive disease was defined as no symptoms or signs of inflammation and a normal ESR for six consecutive months [6]. The definition of clinical inactive disease was chosen with reference to Wallace et al.’s preliminary criteria for inactive disease and clinical remission in juvenile idiopathic arthritis [31].

### WBMRI protocol

A WBMRI was performed at baseline and repeated after one year as part of a clinical routine. Either a 1.5 Tesla Siemens Magnetom Avanto (Erlangen, Germany) or a Philips Achieva dStream (Eindhoven, The Nederland) was used. In all children a coronal whole-body short tau inversion recovery (STIR) sequence with a slice thickness of 5 mm was performed. A coronal T1-weighted sequence was performed in 11/18 children at baseline and in 15/18 children at year-one. Site specific MRI was performed when indicated clinically or radiologically. At baseline, this encompassed sagittal STIR of the spine (*n* = 8), axial STIR of the pelvis (*n* = 8), sagittal STIR of the feet (*n* = 6) and other anatomical regions (*n* = 6). At year-one site specific MRI encompassed sagittal STIR of the spine (*n* = 6), axial STIR of the pelvis (*n* = 10), sagittal STIR of the feet (*n* = 13) and other anatomical regions (*n* = 6).

### WBMRI assessment

The WBMRI examinations were retrospectively evaluated on a dedicated PACS workstation (Agfa Impax, Belgium, version 6.3.1.8000) with 2 K Bracco screens independently by two experienced musculoskeletal radiologists (blinded for review) for the presence and size of any bone lesions. The presence of a lesion was defined as an area of bone marrow hyperintensity compared to surrounding normal bone marrow. All measurements of lesion sizes, except spinal lesions, were performed on the whole body STIR sequences where the largest diameter of hyperintensity lesions was used as a size measure. The size of the spinal lesions was measured on sagittal spinal STIR sequences obtained if there was spinal bone marrow oedema on the coronal STIR images or spinal symptoms. The radiologists were blinded to clinical data, but due to the metabolic effects of pamidronate at the metaphyses it was not possible to blind assessors to pre- and post-treatment imaging.

**Fig. 2:**
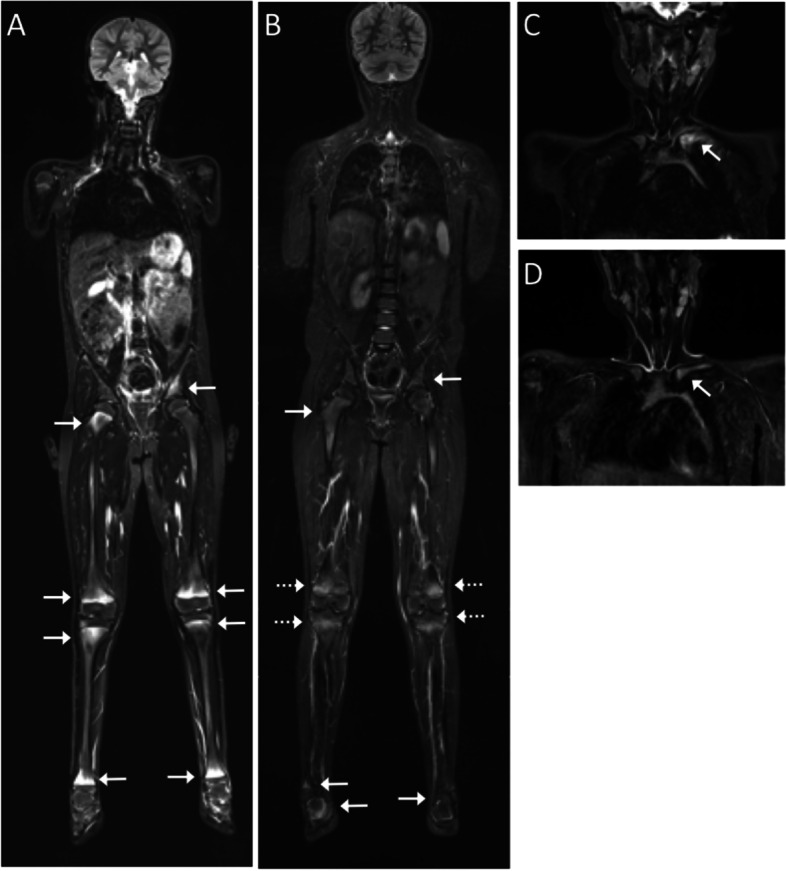
Whole-body MRI following one year of pamidronate treatment. Representative MRI images of a 7-year-old girl diagnosed with chronic non-bacterial osteomyelitis (CNO) and imaging following one year of pamidronate treatment. **A** Pre-pamidronate whole-body magnetic resonance imaging (WBMRI) short tau inversion recovery (STIR), coronal image shows multiple areas of bone marrow edema as signs of radiologically active lesions (RAL) (white thin arrows). **B** Post-pamidronate coronal WBMRI STIR image showed resolution of RAL in the left acetabulum and regression of RAL in the right femur. Assessment of RAL in the distal femur and proximal tibia is challenged by bisphosphonate bands (dotted arrows) **C** Pre-pamidronate and **D** post-pamidronate coronal MRI STIR images of the clavicles showing post-treatment regression of bone marrow edema and surrounding soft tissue edema

At year-one, bisphosphonate induced sclerotic and edematous metaphyseal bands parallel to the growth plates could challenge the assessment of bone lesions, especially in the knee region. This was handled with appropriate window setting (Fig. 2).

### Anatomical distribution of radiologically active bone lesions

The location of bone lesions was divided into seven anatomical regions as shown in Table 2. Besides, the location of tubular bone lesions was assessed in relation to epiphysis, metaphysis, diaphysis and hyper-vascularized growth areas adjacent to apophyses [32–34].

**Table 2:** Distribution of radiologically active bone lesions at baseline and after one year of pamidronate therapy

	Baseline	Year 1	Baseline	Year 1	*P-value*
	RAL n (%)	RAL n (%)	Children n (%)	Children n (%)	
*Head*	*1 (1)*	*1 (2)*	*1 (6)*	*1 (6)*	*-*
Mandibula	1 (1)	1 (2)	1 (6)	1 (6)	
*Chest*	*6 (5)*	*5 (8)*	*6 (33)*	*5 (28)*	*0.5*
Clavicula	3 (3)	3 (5)	3 (17)	3 (17)	
Sternum	1 (1)	0	1 (6)	0	
Scapula*	2 (2)	2 (3)	2 (11)	2 (11)	
*Upper extremities*	*4 (4)*	*0*	*3 (17)*	*0*	*0.13*
Proximal humerus	1 (1)	0	1 (6)	0	
Distal humerus	1 (1)	0	1 (6)	0	
Proximal radius/ulna	1 (1)	0	1 (6)	0	
Distal radius/ulna	1 (1)	0	1 (6)	0	
*Pelvis*	*21 (19)*	*13 (21)*	*10 (56)*	*8 (44)*	*0.06*
Pelvis-ileum*	11 (10)	5 (8)	8 (44)	4 (22)	
Pelvis-pubis	1 (1)	1 (2)	1 (6)	1 (6)	
Pelvis-ischium	2 (2)	3 (5)	2 (11)	3 (17)	
Pelvis-sacrum	7 (6)	4 (7)	6 (33)	4 (22)	
*Distal extremities*	*49 (45)*	*26 (43)*	*16 (89)*	*9 (50)*	< *0.01*
Proximal femur*	10 (9)	6 (10)	7 (39)	3 (17)	
Distal femur	8 (7)	3 (5)	6 (33)	2 (11)	
Proximal tibia*	11 (10)	4 (7)	9 (50)	3 (17)	
Distal tibia*	15 (14)	9 (15)	9 (50)	6 (33)	
Proximal fibula	0	0	0	0	
Distal fibula	5 (4)	4 (7)	4 (22)	3 (17)	
*Feet*	*13 (12)*	*10 (17)*	*8 (44)*	*6 (33)*	*0.55*
Foot-calcaneus *	5 (4)	6 (10)	4 (22)	4 (22)	
Foot- talus*	3 (3)	2 (3)	3 (17)	2 (11)	
Foot-middle	5 (4)	2 (3)	4 (22)	1 (6)	
*Spine*	*16 (15)*	*6 (10)*	*6 (33)*	*3 (17)*	< *0.01*
Cervical vertebra	0	0	0	0	
Thoracic vertebra*	15 (14)	2 (3)	5 (28)	2 (11)	
Lumbar vertebra*	1 (1)	4 (7)	1 (6)	1 (6)	
**Total**	**110 (100)**	**61 (100)**	**-**	**-**	

Distribution of radiologically active lesions (RALs) at baseline and after one year of pamidronate treatment (*n* = 18). Anatomic locations were categorized into seven anatomic regions. Anatomic distribution of RALs was assessed by WBMRI prior to and following one year of pamidronate treatment. RALs are presented as number (and percentage of total RALs) prior to pamidronate (total 110 RAL) and year one (total 61 RAL). RALs are also presented as number of children (and percentage of 18 children) with RALs at the different anatomic locations. McNemar´s test was used to compare distribution of RALs in children during pamidronate treatment. P-value < 0.05 was considered significant. *Indicates anatomic locations with new RAL at year one

### Definitions of parameters of interest

Radiologically active lesions (RALs) were defined as areas of manifest local increased signal intensity in the bone marrow on STIR images, and concomitant periosteal edema and/or soft tissue edema was defined as active extramedullary changes. Cortical thickening or bone-expansion/hyperostosis were defined as chronic hyperostotic changes and deformation of vertebra was interpreted as sign of chronic spinal involvement. The size of all RALs and the size of the largest RAL for each patient (RALmax) were recorded, and the height of affected vertebral bodies was measured with regard to vertebral collapse.

### Modified radiological index for non-bacterial osteitis (mRINBO)

A composite quantification of the above stated parameters assessed by WBMRI was elaborated in accordance with the RINBO method (Table 1) [35]. A modification of the RINBO score regarding RALmax seems needed because all our RALmax lesion sizes at baseline and follow-up were within the range of minor and average measures in the original RINBO score giving one and two points, respectively, and the possibility of a three-point scoring value was missed. This resulted in a modified (m)RINBO quantification used for all WBMRI (Table 1). Also, the metatarsal bones were excluded from the assessment due to a general poor visualization on coronal images, and known difficulties regarding differentiation of metatarsal CNO lesions from non-specific hyperintensities in the feet [36].

### Statistical analysis

Normally distributed data were presented as mean (standard deviation, sd) and data not normally distributed were presented as median [interquartile range, IQR]. Comparison between baseline and year one was made using Wilcoxon signed rank test for categorical data and McNemars test for paired dichotomous data. A bootstrapping method was used to determine changes in the size of all RALs. The two-way random effect model for absolute agreement was used for testing the individual intraclass correlation coefficient (ICC) regarding the presence of CNO lesions. The mean values for lesions sizes were used for the mRINBO scoring. *P*-values < 0.05 were considered statistically significant. The statistical analyses were performed using Stata 14 (StataCorp).

## Results

### Patients’ characteristics

Eighteen children fulfilled the study criteria (Fig. 1). The time from first symptoms to diagnosis was median 11 [IQR, 5–24] months. All children were treated with naproxen initially. A WBMRI was performed mean 1.2 (sd, 0.7–1.7) months prior to initiation of pamidronate treatment and follow-up WBMRI was performed after a mean of 12 (sd, 11.1–13.3) months. Pamidronate treatment was initiated median 5 [IQR, 0.7–9.2] months after the diagnosis was obtained. None of the children received conventional synthetic DMARDs or glucocorticoids during the study period.

From baseline to year-one ESR decreased from median 14 [IQR, 9–23] mm/h to 7 [IQR, 3–12] mm/h (*p* = 0.01). One of 9 children tested was HLA-B27 positive. A bone biopsy was performed in 13/18 children, revealing only non-specific inflammatory changes.

### Anatomical distribution of radiologically active bone lesions

The most common locations of RALs were in the long tubular bones of the lower extremities, pelvis and spine (Table 2). The majority of RALs in the tubular bones and the pelvis were located to hyper-vascularized areas adjacent to growth zones such as metaphyseal cartilage plates and cartilage at apophyses, especially at the trochanter region. Thus, at baseline 5 of 10 RALs in the proximal femur were located to the apophyseal region at the trochanter and five at the growth plate. Most of the 21 pelvic RALs were also located adjacent to the growth areas; five occurred at the triangular cartilage, two at the ischiopubic synchondrosis, six at the acetabular roof, seven in the sacrum adjacent to the SI-joint and one in the ischium (Table 2, Fig. 2).

### Number of radiological active bone lesions

**Fig. 3:**
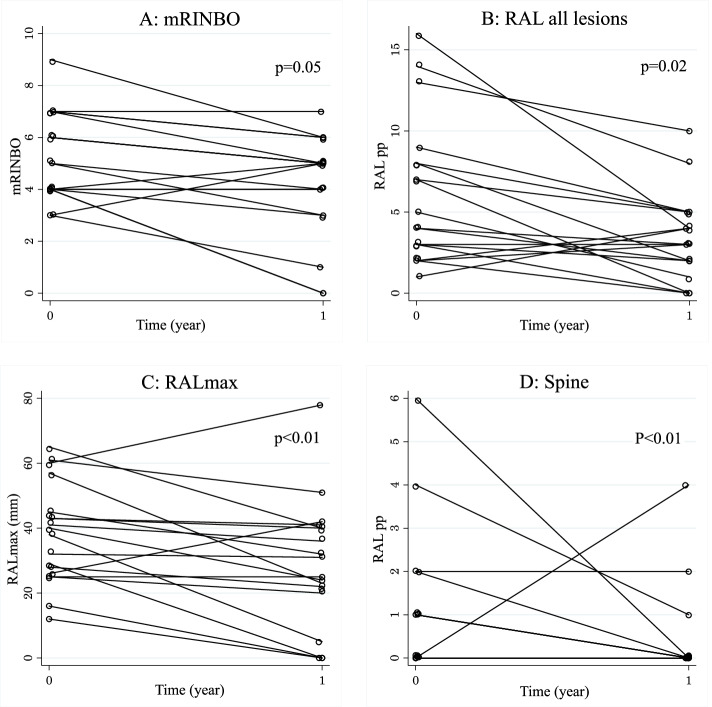
Changes in radiological disease activity following one year of pamidronate treatment. Radiological response to pamidronate treatment in children diagnosed with severe CNO (*n* = 18). WBMRI was assessed prior to pamidronate treatment and following one year of treatment. **A** Changes in the modified radiological index for non-bacterial osteitis (mRINBO), *p* = 0.05 **B** Changes in total number of radiologically active lesions (RAL) per patient, *p* = 0.02 **C** Changes in the size of the largest RAL for each patient (RALmax) *p* < 0.01. **D** Changes in the number of RAL for each patient in the spine, *p* < 0.01. Comparison between baseline and year one was made using Wilcoxon signed rank test

At baseline, the total number of RALs was 110 with a median of 4.5 [IQR, 3–8] RALs per patient. The RALs were symmetrically located in 17/18 children. From baseline to year-one, 67/110 (61%) RALs resolved, and 3/18 children became without signs of radiological activity by WBMRI (Fig. 3). However, 18 new RALs appeared in 7/18 (39%) of the children at year-one. The total number of RALs at year-one was 61 with a median of 3 [IQR, 2–5] RALs per patient, which was a significant decrease of the total number of RALs per patient from baseline to year-one (*p* = 0.02) (Table 2, Fig. 3).

Spinal involvement encompassed 16 vertebral RALs in 6/18 (33%) of the children at baseline. At year-one 14/16 vertebral lesions had resolved (*p* < 0.01) (Fig. 3), but one child without vertebral lesions at baseline developed four new asymptomatic lumbar vertebral RALs (Table 2, Fig. 3 and supplementary file S1). Vertebral deformation (collapse) was observed in 4 children at baseline with no improvement during pamidronate therapy. There was no difference in ESR in children with or without vertebral fractures.

### Size of radiological active lesions

At baseline, the median size of RALs (*n* = 110) was 21 [IQR, 14–29] mm. The size of these RALs decreased to 0 [IQR, 0–22] mm at year-one, *p* < 0.01 (Supplementary file S2). However, due to the new RALs developing from baseline to year-one the median size of all RALs at year-one (*n* = 61) was 22 [IQR, 5–31] mm and thus did not change from baseline. However, the median size of RALmax decreased from 39 [IQR, 26–45] mm at baseline to 28 [IQR, 20–40] mm at year-one (*p* < 0.01) (Fig. 3). The largest RALmax at baseline was 65 mm and at year-one 78 mm, both were symptomatic and located at the pelvic triangular cartilage.

### Extramedullary affection

At baseline, extramedullary active inflammatory changes were observed in relation to 9 of the 110 (8%) RALs in 7 children, being located to the mandible (*n* = 1), the clavicle (*n* = 3), os ischium (*n* = 1) and the proximal tibia (*n* = 1). Except from the lesion in the tibia all extramedullary edematous changes had resolved year-one (Fig. 2). New extramedullary changes were not observed from baseline to year one. Hyperostosis was observed in relation to the clavicular and mandibular RALs with regression during treatment.

### Modified RINBO score

The composite mRINBO score taking the number of RALs, RALmax, extramedullary and spinal lesions into account, decreased from a median of 5 [IQR, 4–7] at baseline to a median of 4 [IQR, 3–5] at year-one, *p* = 0.05 (Fig. 3).

### Interrater agreement

The observer difference regarding the size of RALs was maximum 5 mm and the mean sizes were therefore used in the estimation of mRINBO. Disagreements regarding the presence of active bone lesions occurred only in relation to two lesions (one in femur, one in pelvis). This discordance was solved by consensus. ICC was 0.997 (0.991 – 0.999) indicating an excellent reliability.

### Clinical disease activity

Symptoms were reported in relation to all anatomic RAL subgroups. However, from clinical records it was not always possible to assess if each RAL was symptomatic. At year-one, 8/18 (44%) children had clinical inactive disease and 9 of the remaining 10 children reported clinical improvement. There was no difference between children with clinically inactive disease and children with clinically active disease at year-one regarding the number of RALs per patient and mRINBO.

The median number of RAL was 3 [IQR, 1–3] per patient and the median mRINBO score was 5 [IQR, 2.5–5.5] in children with clinically inactive disease year-one, whereas the median number of RAL was 3.5 [IQR, 2–5] per patient and the median mRINBO score was 4.5 [IQR, 3–5] in children with clinically active disease.

## Discussion

Modifications of the original radiological index for non-bacterial osteitis (RINBO) [35] regarding the size of RALmax and exclusion of RAL in the metatarsal bones were implemented, and the modified mRINBO was used for standardized reporting and quantification of WBMRI findings in children with CNO to evaluate the effect of pamidronate therapy. mRINBO was assessed initially and after one year of pamidronate therapy.

We observed an improvement in radiological disease activity assessed by mRINBO following one-year therapy. From baseline to year-one the mRINBO decreased from a median of 5 [IQR, 4–7] to a median of 4 [IQR, 3–5] (*p* = 0.05) driven by a significant decrease in the number of RALs per patient, a decrease in the size of RALmax and resolution of extramedullary changes in all but one bone-lesion situated in the tibia. Signs of reduced radiological activity occurred especially in the pelvis, in the long tubular bones of the lower extremities and in the thoracic spine. These findings are in concordance with prior studies in CNO, that report similar response to pamidronate, when radiological disease manifestations are assessed by MRI [21, 23, 29, 37].

When assessing the size of RALmax we observed, that the largest RAL at baseline was 65 mm and the median size of RALmax was 39 [IQR, 26–45] mm and at year-one, the largest RAL was 78 mm and the median size of RALmax was 28 [IQR, 20–40] mm. In the original RINBO study the largest RAL was considerable larger being 265 mm (mean 64 ± 55 mm) [2]. The size of RAL depends on disease activity, but also on the size of the bone involved, implying that the age of child and the anatomic location can influence the size of RAL and RALmax. CNO lesions vary considerably in size; the largest lesions often occurring in the pelvis and future studies may have to prove that the maximal size of RAL is an important feature of disease activity. Adjustment using relative measurements of RAL taking the various bone sizes into account may also be considered in future studies [38].

The anatomical distribution of bone lesions was in agreement with previous studies in CNO [4, 29]. Metatarsal bone lesions were omitted because assessment of metatarsal lesions revealed that they were often clinically silent. Radiological CNO findings in the feet have previously been reported in a WBMRI study of 53 children with CNO. This study also showed a mismatch between radiological and clinical findings in the metatarsal bones [5]. Furthermore, metatarsal bone lesions are difficult to assess by coronal WBMRI and difficult to differentiate from non-specific hyperintensities in the feet which may be a normal finding in children [36]. Therefore, if lesions in the forefoot are considered of clinical significance, it is recommended to perform site specific MRI assessment or plain radiographs, which may disclose characteristic CNO changes in the feet, including osteolysis, osteosclerosis and new bone formation [9, 39].

In CNO, WBMRI is often used for diagnosis and may be used as follow-up [12]. An advantage of the mRINBO score is that it follows a systematic quantitative approach and results in a summary patient score. In this study, we assessed changes in radiological disease activity in children with CNO treated with pamidronate for one year. However, mRINBO may be used to assess radiological changes independently of the therapy used. So far, assessment of changes in radiological disease activity in children with CNO has previously predominantly been descriptive, mainly reporting the number of bone lesions and changes in bone marrow edema based on expert opinion [3, 8, 21, 23, 24, 30, 37, 40–44]. *Panwar *et al*.* report changes in radiological disease activity in predefined anatomical location based on a WBMRI scoring system. This system encompass the number, relative size and signal intensity of active CNO lesions as well as soft tissue inflammation and structural lesions in the form of osseous hyperostosis and vertebral collapse [29]. *Zhao *et al*.* suggest another consensus-driven method for semiquantitative grading of the various CNO lesions by WBMRI, ´chronic nonbacterial osteomyelitis magnetic resonance imaging scoring´ (CROMRIS). This method include both active and structural changes and has an advantage of using a relative size measure of the bone lesion in relation to the bone sizes in affected children [38]. However, these methods do not describe a summary patient score and have to our knowledge not been tested in longitudinal studies, probably because further studies are needed to determine the weighting of each variable.

Monitoring CNO activity is complicated by the fluctuating disease course with spontaneous development of new RALs [3, 39], also during treatment [8, 30]. The mRINBO has the advantage of following a systematic quantitative approach reporting the accumulated radiological disease activity in each individual with CNO. Also, the mRINBO quantification is relatively easy to obtain and takes the important features of CNO into account, considering anatomical locations in addition to lesion number and size. Some anatomical locations may be considered to imply an increased risk such as spinal lesions as they can be osteolytic and lead to vertebral collapse [7, 8, 45]. We observed a statistically significant improvement in the number of spinal RAL, but it was not possible to show improvements in vertebral collapse during one year of pamidronate treatment as has been show in a previous case studies of long lasting pamidronate treatment up to 41 months [8, 19].

Information on clinical disease activity was assessed retrospectively and therefore not acquired in a standardized manner. We were not able to show a correlation between changes in mRINBO and clinical disease activity. A lack of correlation between clinical and radiological disease activity and occurrence of asymptomatic CNO lesions have previously been reported [2, 3]. Our definitions on clinical disease activity were chosen with reference to Wallace et al.’s preliminary criteria for inactive disease and clinical remission in juvenile idiopathic arthritis [31]. Previously, PedCNO has been suggested to describe improvements in disease activity in CNO in prospective studies [3]. PedCNO is a clinical/radiological composite score, that refers to a set of 5 outcome variables in CNO: ESR, number of radiological active bone lesions, severity of disease estimated by the physician, severity of disease estimated by patient or parent, and the Childhood Health Assessment Questionnaire (CHAQ). However, PedCNO has a limitation as the radiological measure only encompass the number of radiological active bone lesions and not lesion size, site, extramedullary changes or chronic changes. In case studies, clinical disease activity has also been assessed as pain on a visual analogue scale [21, 42, 43, 46] and CHAQ [43, 46] and both measures have been shown useful to describe changes in clinical disease activity in CNO.

There were several limitations in our study. First, the size of the study population is limited and the study cohort is non-controlled. It may be possible that the radiological improvement observed can be due to an innate disease course. Secondly, WBMRI was performed on two different MRI scanners and the protocol used did not always include a T1-weighted sequence. Systematically performed T1-weighted sequences might have contributed to a higher sensitivity and specificity in assessing bone lesions than STIR alone. Thirdly, the study population was selected based on inclusion and exclusion criteria regarding CNO severity and uniform imaging regarding modality and timing. Children were treated with pamidronate according to a local treatment algorithm. Six children who received TNF-α inhibitor treatment were excluded from the study as they were mainly treated with bDMARDs due to inflammatory comorbidity and not due to CNO. Exclusion of these children might have led to bias. Also, exclusion of children not fulfilling the imaging criteria might have led to bias as children not examined by WBMRI might have a milder clinical disease course.

## Conclusion

In CNO, children receiving pamidronate treatment, radiological disease activity decreased, when assed by mRINBO, indicating a therapeutic effect. The mRINBO score may be a potential method for standardized reporting and quantification of WBMRI findings and radiological changes in children with CNO. However, further development is needed to investigate if mRINBO is a feasible and valid scoring tool. This will include studies of RALmax including considerations using relative size measurements of bone lesions and interrater agreement and reliability analyses in a larger representative cohort. This study also showed, that a standardized WBMRI protocol including coronal STIR and T1-weighted imaging in addition to sagittal imaging of the spine must be recommended to obtain uniform scoring of active and structural radiological changes in CNO.

## Supplementary Information

**Additional file 1: Image supplementary S1.** MRI images of a 9-year-old girl diagnosed with CNO involving the spine. Images before and after one year of pamidronate treatment. (A, B) Pre-pamidronate, coronal and sagittal short tau inversion recovery (STIR) images show bone marrow edema in vertebra Th4,5,6 and Th8, most pronounced in Th6. Vertebral deformation was detected in Th1,4,5,6 and Th8. (C, D) Post-pamidronate, coronal and sagittal STIR images show resolution of bone marrow edema in the involved vertebra, but persistence of vertebral deformities. **Supplementary S2.** Size in radiologically active bone lesions at baseline and their size after one year of pamidronate therapy.

## Data Availability

The datasets used and/or analyzed during the current study are available from the corresponding author on reasonable request.

## Abbreviations

CHAQ: Childhood Health Assessment Questionnaire
CNO: Chronic non-bacterial osteomyelitis
DMARD: Disease modifying anti-rheumatic drug
ESR: Erythrocyte sedimentation rate
mRINBO: Modified radiological index for non-bacterial osteitis (mRINBO)
NSAIDs: Non-steroidal anti-inflammatory drugs
RAL: Radiologically active bone lesions
RALmax: Maximal size of radiologically active bone lesions
RINBO: Radiological index for non-bacterial osteitis (mRINBO)
STIR: Short tau inversion recovery
WBMRI: Whole-body magnetic resonance imaging
