# Supplementary material for: Standardized reporting and quantification of whole-body MRI findings in children with chronic non-bacterial osteomyelitis treated with pamidronate
Source: Pediatr Rheumatol Online J. 2022 Oct 1;20:85. doi: 10.1186/s12969-022-00746-y (PMC9526975; doi:10.1186/s12969-022-00746-y)
Supplement: Supplementary file 1 — Additional file 1: Image supplementary S1. MRI images of a 9-year-old girl diagnosed with CNO involving the spine. Images before and after one year of pamidronate treatment. (A, B) Pre-pamidronate, coronal and sagittal short tau inversion recovery (STIR) images show bone marrow edema in vertebra Th4,5,6 and Th8, most pronounced in Th6. Vertebral deformation was detected in Th1,4,5,6 and Th8. (C, D) Post-pamidronate, coronal and sagittal STIR images show resolution of bone marrow edema in the involved vertebra, but persistence of vertebral deformities. Supplementary S2. Size in radiologically active bone lesions at baseline and their size after one year of pamidronate therapy. [file 12969_2022_746_MOESM1_ESM.docx]

**Supplementary material**

**Image supplementary (S1)**

**
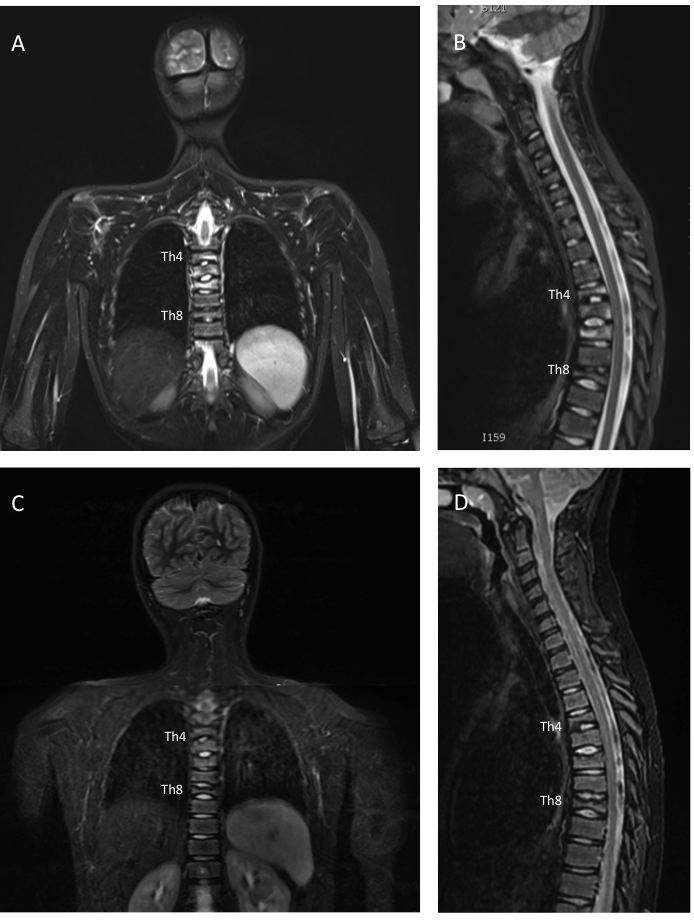
**

**S1:** Whole-body MRI images of the spine following one year of pamidronate treatment

MRI images of a 9-year-old girl diagnosed with CNO involving the spine and images before and after one year of pamidronate treatment. (A, B) Pre-pamidronate, coronal and sagittal short tau inversion recovery (STIR) images show bone marrow edema in vertebra Th4,5,6 and Th8, most pronounced in Th6. Vertebral deformation was detected in Th1,4,5,6 and Th8. (C, D) Post-pamidronate, coronal and sagittal STIR images show resolution of bone marrow edema in the involved vertebra, but persistence of vertebral deformities.

**Supplementary 2 (S2)**

| **S2**. Size in radiologically active bone lesions at baseline and their size after one year of pamidronate therapy | | | | | |
| --- | --- | --- | --- | --- | --- |
| Lesion site (mm) | | Baseline | Year 1 | p-value |  |
| *Head* | |  |  |  |  |
|  | Mandibula | 43 | 41 | 0.25 |  |
| *Chest* | | *24 [20-32]* | *21 [0-28]* | *0.11* |  |
|  | Clavicula | 24 | 25 | 0.29 |  |
|  | Sternum | 20 | 0 | 0.32 |  |
|  | Scapula | 20 | 19 | 0.17 |  |
| *Long bones of upper extremities* | | *15 [11-22]* | *0* | *<0.01* |  |
|  | Proximal humerus | 18 | 0 | 0.32 |  |
|  | Distal humerus | 26 | 0 | 0.32 |  |
|  | Prox. radius/ulna | 11 | 0 | 0.32 |  |
|  | Distal radius | 11 | 0 | 0.32 |  |
| *Pelvis* | | *28 [17-45]* | *10 [0-23]* | *<0.01* |  |
|  | Pelvis-ileum | 41 | 44 | 0.02 |  |
|  | Pelvis-pubis | 41 | 36 | 0.32 |  |
|  | Pelvis-ischium | 25 | 17 | 0.18 |  |
| Pelvis-sacrum | | 26 | 15 | 0.09 |  |
| *Long bones of lower extremities* | | *20 [14-34]* | *0 [0-25]* | *<0.01* |  |
|  | Proximal femur | 18 | 5 | 0.11 |  |
|  | Distal femur | 26 | 0 | 0.01 |  |
|  | Proximal tibia | 19 | 0 | <0.01 |  |
|  | Distal tibia | 21 | 5 | 0.04 |  |
|  | Distal fibula | 18 | 17 | 0.69 |  |
| *Feet* | | *21 [11-23]* | *20 [0-29]* | *0.11* |  |
|  | Foot- calcaneus | 29 | 13 | 0.21 |  |
|  | Foot- talus | 23 | 0 | 0.11 |  |
|  | Foot- middle | 13 | 0 | 0.06 |  |
| *Spine* | | *21 [11-23]* | *0 [0-0]* | *<0.01* |  |
|  | Cervical vertebra | 0 | 0 | - |  |
|  | Thoracic vertebra | 21 | 0 | 0.003 |  |
|  | Lumbar vertebra | 21 | 5 | 0.32 |  |
| **Total** | | **21 [14-29]** | **0 [0-22]** | **<0.01** |  |
| Size of RALs at baseline (n=110) and after one year pamidronate therapy. Anatomic locations were categorized into 7 anatomic regions. Total resolution was observed in 67/110 RALs. Data are presented as median and interquartile range. Statistical significance was found using Wilcoxon signed-rank test in lesion site and bootstrapping method was used to determine changes in RAL size in anatomic regions. P-values < 0.05 were considered significant | | | | | |
